# Indigenous population and major depressive disorder in later life: a study based on the data from Longitudinal Ageing Study in India

**DOI:** 10.1186/s12889-022-14745-x

**Published:** 2022-12-03

**Authors:** Rashmi Rashmi, Shobhit Srivastava, T. Muhammad, Manish Kumar, Ronak Paul

**Affiliations:** International Institute for Population Sciences, Mumbai, 400088 India

**Keywords:** Social groups, Indigenous population, Depression, Older adults, India

## Abstract

**Background:**

Existing evidence suggests that the indigenous older population who live with their families and friends might experience lesser depressive symptoms due to better emotional support and well-being. The present study aimed to investigate the differentials in the prevalence of the major depressive disorder among tribal and non-tribal older populations in India and to explore the contribution of socio-demographic, health-related, and household factors in such disparities.

**Methods:**

A cross-sectional study was conducted using data from the Longitudinal Aging Study in India (2017–18). The analytical sample included 30,637 older adults, among whom 5,025 and 25,612 belonged to the Scheduled Tribe (ST) and non-Scheduled Tribe (non-ST) social groups, respectively. Major depressive disorder assessed by the Composite International Diagnostic Interview short-form (CIDI-SF) scale was the outcome variable. Descriptive statistics, bivariate and multivariable regression and, decomposition analyses were conducted.

**Results:**

About 4.8% and 8.9% of older adults from the ST and non-ST social groups had major depression. For both tribal and non-tribal groups, older adults who were unmarried, dissatisfied with living arrangements, and those who faced lifetime discrimination were at increased risk of major depression. Findings from differences due to characteristics (E) revealed that if the regional differences were minimized, it would decrease the ST-non-ST gap in major depression by about 19.6%. Similarly, equal self-rated health status and chronic conditions among ST and non-ST groups would decrease the gap in major depression by almost 9.6% and 7.9%, respectively. Additionally, an equal status of Instrumental Activities of Daily Living (IADL) and Activities of Daily Living (ADL) among older adults would decrease the gap in major depression by about 3.8% and 3% respectively. Also, findings from differences due to coefficients (C) revealed that if older adults from the ST group had the same status of ADL as of older adults from the non-ST group, it would decrease the gap in major depression by about 11.8%.

**Conclusion:**

The findings revealed a greater prevalence of major depression in older adults belonging to the non-ST group than the ST group. For both tribal and non-tribal groups, older adults who were unmarried, dissatisfied with living arrangements, and those who faced lifetime discrimination were at increased risk of major depression and these factors along with health-related variables contributed to significant ST-non-ST gap in depression, advantageous to tribal population; suggesting further research on the coping mechanisms of mental illnesses among indigenous population in India.

## Introduction

With about 3.8% depressed population worldwide, a common mental health issue, depression, is slowly sneaking into the lives of people of all ages [1]. According to the World Health Organization, depression results from persistent sadness and a loss of interest in one’s usual activities, accompanied by an inability to carry out daily activities for at least two weeks [1]. Even though depression is preventable, studies show that more than 75% of individuals do not receive treatment in low-and-middle-income countries [2]. Such negligence is mostly due to the misconception that depression is a part of the ageing process, making the 60 years and above population more vulnerable [3].

India, one of the lower-middle-income countries, is no exception [3]. According to the National Mental Health Survey 2015–16, one in every 20 Indians suffers from depression [4]. Although the most affected age group were those aged 15–49 years, the continuous Indian demographic transition has generated health concerns for the aged people in recent years. According to the Longitudinal Aging Study in India, about 8.3% of older adults aged 60 years and above experienced major depression based on the Short Form Composite International Diagnostic Interview (CIDI-SF) scale [5]. Depression triggers many health problems like obesity, diabetes, heart disease, physical activeness, and vice-versa [6–9]. A complex interaction of social, psychological, and biological factors further triggers depression in an individual [10–12].

In India, inequities in health and healthcare utilization between advantaged and marginalized groups persisted throughout the years [13]. Extant research has demonstrated social status as a significant predictor of health disparities in India [14–17]. A study from the World Health Organization’s Survey of Global Ageing and Adult Health (WHO-SAGE) indicated higher mental health issues among Scheduled Castes and Muslims than among Higher caste Hindus [18]. Ample evidence shows the depression vulnerability of the tribal population in the Indian context [18–21]. Changing lifestyle, beliefs, and community living, moving to urban spaces, and triggering adverse life events like morbidity, unemployment, bereavement, stress, and psychological trauma are the emerging factors predisposing to mental health issues like depression [22].

Several studies have associated depression with lower socioeconomic status; however, a study indicated that emotional well-being (associated with depression) and life evaluation (associated with life satisfaction) have different correlates, which are often considered the same mistakenly [23]. For instance, there is a long-run question of whether money buys happiness or not. A study from the US shows that emotional well-being is highly associated with the positive and negative emotions created by circumstances like caring for a sick relative and spending time with family and friends. In contrast, life evaluation was highly associated with socioeconomic status [23]. The study concludes that a high income can bring life satisfaction but not happiness. According to these findings, the indigenous older population living with their families and friends might experience lesser depressive symptoms due to better emotional well-being. However, the long negligence of mental health issues in the tribal population also raises concern on their current situation.

Thus, the present study aimed to investigate differentials in the prevalence of depression among the tribal and non-tribal populations in India, primarily focusing on the vulnerable older population. Moreover, we explored the contribution of socio-demographic, health-related, and household factors to depression disparity across tribal and non-tribal older adults in India.

## Materials and methods

### Data source

The present study utilized the data from the baseline wave (wave 1) of the Longitudinal Aging Study in India (LASI), conducted during 2017–18. The LASI is a biennial panel survey representative of the older and elderly population age 45 and above for India and its states and union territories. The LASI wave 1 interviewed 42,949 households and 72,250 individuals aged 45 + years and their spouses across India’s 30 states and six union territories. It adopted a multistage stratified area probability cluster sampling design: a three-stage sampling design in rural areas and a four-stage sampling design in urban areas.

The LASI is a multi-topic, nationally representative, a large-scale survey conducted jointly by the International Institute for Population Sciences (IIPS), the Harvard T.H. Chan School of Public Health (HSPH), and the University of Southern California (USC). It is the Indian version of the Health and Retirement Studies (HRS). It provides vital information on chronic health conditions, symptom-based health conditions, demography, functional and mental health, household economic status, healthcare utilization, health insurance, work, employment and retirement, and life expectations of 45 years and above participants with their spouses, irrespective of age. Further details regarding the sample design, survey instruments, fieldwork, data collection and processing, and response rates are publicly available in the report [5].

### Sample and participants

The Indian constitution divides the social groups into three broad categories- Scheduled Tribes [ST], Scheduled Caste [SC], and Other Backward Classes [OBC]. Those who do not fall in these groups are categorized as “Others”. Both SC and ST include mostly socially backward groups. OBC is socially and economically backward but has a much better situation than SC/ST. The ST group, which has a predominantly tribal population, comprises 8.6% of India’s population. Traditionally, these tribal groups reside away from mainstream Indian society and consume nature-based resources for daily health requirements [24, 25]. We have recoded the social group variable into ST (tribal or indigenous) and Non-ST groups in the present study.

In this study, we utilized the sample of 31,464 Indian older adults aged 60 years and above (5,173 belonging to the ST and 26,291 belonging to the non-ST groups). 827 older adults whose depression information was not available in the data were excluded from this study. Therefore, the analytical sample was 30,637 older adults, among whom 5,025 and 25,612 belonged to the ST and Non-ST social groups, respectively.

### Outcome variable

The main outcome variable was major depression among older adults categorized as “Depressed” and “Not depressed”.

Major depression was assessed in LASI using the successfully tested and widely-utilized Composite International Diagnostic Interview short-form (CIDI-SF) scale that assesses psychiatric depression [5, 26]. There are ten questions and the response for all the items (except items “2” and “3”) were in binary format (i.e., “No” coded as 0, and “Yes” coded as 1). Again, those individuals who felt sad, blue or depressed “all day long” or “most of the day” were coded as “Yes”, else they were coded as “No”. Equivalently, individuals who felt sad, blue or depressed “every day” or “almost every day” were coded as “Yes”, else, they were coded as “No”. The scale had a Cronbach’s α reliability coefficient of more than 0.6. Next, we summated the ten items to obtain the CIDI-SF depression scale, with scores ranging from 0 to 10. Based on extant literature, Indian older adults with a score of 5 and more were categorized as “Depressed”, and those with a score of 4 and less were classified as “Not depressed” [27, 28].

### Explanatory variables

Based on existing research, we included the following socio-demographic, health-related, and household characteristics in this study. The socio-demographic characteristics of older adults include the following –

1. Age group was coded as (oldest-old, old-old, young-old). The young-old, old-old and oldest-old comprise older adults aged 60 to 69 years, 70 to 79 years, and 80 years or more, respectively.
2. Gender was coded as (male, female).
3. Marital status was coded as (currently married, currently not married).
4. Status of work was coded as (never worked, currently not working, currently working, retired).
5. The level of education was coded as (no formal education, upto primary, secondary and above).
6. Social participation was coded as (socially active, socially inactive). LASI collected information on whether a person goes out of the house for eating, goes outdoors for relaxing, plays indoor games, plays outdoor games or exercises, visits relatives, attends cultural events, attends religious functions, attends group meetings, reads books, newspaper and magazines, watches television or listens to the radio, use a computer for electronic communication. Older adults who undertook any of the above social activities were classified as “socially active” and otherwise were classified as “socially inactive”.
7. The importance of religion was coded as (not important, very important).
8. Living arrangement satisfaction was coded as (satisfied, neutral, not satisfied).
9. Received ill-treatment was coded as (no, yes) within one year from the interview.
10. Faces discrimination in life was coded as (no, yes).
  Next, the health-related characteristics of the older adults include –
11. Self-rated health was coded as (good, average, poor).
12. Chronic morbidity status was coded as (no condition, single condition, multiple conditions). LASI collected information on whether an older adult was ever diagnosed with hypertension or high blood pressure, diabetes or high blood pressure, cancer or malignant tumour, chronic lung diseases, chronic heart diseases, stroke, bone or joint diseases, any neurological or psychological problems, high cholesterol. Individuals having no diseases, any one of the diseases and two or more diseases were categorized into “no condition”, “single condition”, and “multiple conditions”.
13. Physical activity status was coded as (physically inactive, physically active). Physical activity status was assessed based on WHO guidelines on physical activity for 18 years and above [29]. Older adults who performed at least 75 min of vigorous-intensity physical activity or at least 150 min of moderate-intensity physical activity in a day or a combination of both were classified as “Physically active” and otherwise were classified as “Physically inactive”.
14. Difficulty in Activities of Daily Living (ADL) was coded as (no difficulty, faces difficulty). Information was collected on whether a person had difficulty in dressing, walking across a room, eating, going in or out of bed, bathing and using the toilet, respectively. During the interview, older adults who faced difficulty in any one of the activities for more than three months were categorized as “faces difficulty”. Those who did not have difficulty in any activities were included in the “no difficulty” category.
15. Difficulty in Instrumental Activities of Daily Living (IADL) was coded as (no difficulty, faces difficulty). LASI obtained information on whether an individual had difficulty in the following seven instrumental activities of daily living: preparing a hot meal, shopping for groceries, making telephone calls, taking medications, doing work around the house or garden, managing household finance and getting around or reaching an unfamiliar place. During the interview, those who faced difficulty in any one of the activities for more than three months were categorized as “faced difficulty”. Else they were included in the “no difficulty” category.
16. Covered by any health insurance was coded as (yes, no).
  Further, the household-related characteristics are –
17. Monthly per capita consumption expenditure was coded as (MPCE) quintile of household (poorest, poorer, middle, richer, richest).
18. Religion was coded as (Hinduism, Islam, Others).
19. The place of residence was coded as (Urban, Rural).
20. The country region that a household is situated (Southern, Northern, Central, Western, Eastern, North-eastern). We constructed the country regions by including the erstwhile 29 states and six union territories of India into six categories based on administrative similarity. The northern part includes Chandigarh, Delhi, Haryana, Himachal Pradesh, erstwhile Jammu and Kashmir, Punjab, Uttaranchal and Rajasthan. The north-eastern region includes Arunachal Pradesh, Assam, Manipur, Meghalaya, Mizoram, Nagaland, Sikkim and Tripura. The central area consists of Madhya Pradesh and Chhattisgarh. The eastern zone consists of Bihar, Jharkhand, Odisha and West Bengal. The western region comprises Dadra and Nagar Haveli, Daman and Diu, Goa, Gujarat and Maharashtra. The southern part includes Andhra Pradesh, Karnataka, Kerala, Pondicherry and Tamil Nadu.

### Statistical methods

The present study applied descriptive statistics, bivariate and multivariable regression and decomposition analyses to find out the results. Using the chi-square test for association, the bivariate analysis looked at the unadjusted link between major depression and health-related, socio-demographic, and household variables. The multivariate association was presented using average marginal effects (AME). Multivariable models did not violate the assumptions of multicollinearity.

We also applied the nonlinear multivariate decomposition method to determine the role of explanatory factors in the social divide in major depression among older adults in India. The ST-non-ST disparity in major depression is decomposed in both an overall and specific. The divide in major depression is decomposed into an endowments or features (E) component and a coefficients or effects (C) component in the overall decomposition. The contribution of each health-, and socio-demographic-, and household-related explanatory factor is dissected in-depth for each endowment and coefficient component of the difference in major depression among tribal and non-tribal groups. The endowments component (E) shows how difference in major depression would change if the distribution of older adults from the ST and non-ST groups for each category of the explanatory variable were similar. Suppose older adults from ST and non-ST groups were equally likely to fall into one of the explanatory variable’s categories. In that case, the coefficients component (C) indicates the expected change in the disparity. Decomposition models may be used to get various estimates for different reference categories of categorical data. We provide normalized decomposition estimates to solve this “identifying difficulty.” STATA software version 13 was used for all the statistical calculations in this study.

All methods were carried out in accordance with relevant guidelines and regulations.

## Results

### Descriptive statistics

Table 1 represents the distribution of ST and Non-ST older adults by socio-demographic, health-related and household characteristics in India. About 4.8% and 8.9% of older adults from ST and non-ST social groups had major depression. About 14.2% of older adults from ST category and 27.5% of older adults from non-ST category never worked. About 73.6% and 55.1% of older adults from ST and non-ST categories had no formal education, respectively. Nearly 29.7% and 20.5% of older adults from ST and non-ST categories did not give importance to religion, respectively. About 4.4% of older adults from ST category and 5.2% of older adults from non-ST category were not satisfied with their living arrangements. Almost 17.6% and 23.6% of the older adults from ST and non-ST categories had poor self-rated health, respectively. About 10.7% of older adults from ST category and 25.0% of older adults from non-ST category had multiple chronic conditions. Almost 38.5% and 26.7% of older adults from ST and non-ST categories were physically activity, respectively. Almost 19.5% & 43.6% of older adults from ST category and 23.2% & 48.0% of older adults from non-ST category had difficulty in ADL and IADL, respectively.

**Table 1:** Distribution of ST and Non-ST older adults by socio-demographic, health-related and household characteristics in India 2017–18

Characteristics	Older adults (60 + years)			
	**ST older adults**		**Non-ST older adults**	
	**N**	**WC %**	**N**	**WC %**
**Depression status**				
Not depressed	4,857	95.2	23,650	91.1
Depressed	168	4.8	1,962	8.9
**Age group**				
Oldest-old	530	7.9	2,597	11.0
Old-old	1,384	28.6	7,459	30.0
Young-old	3,111	63.6	15,556	59.0
**Gender**				
Male	2,371	45.1	12,311	47.4
Female	2,654	54.9	13,301	52.6
**Current marital status**				
Currently married	3,149	61.3	16,367	61.9
Currently not married	1,876	38.7	9,245	38.1
**Status of work**				
Never worked	1,096	14.2	7,444	27.5
Currently not working	1,605	35.5	8,976	35.8
Currently working	1,964	45.3	6,925	29.1
Retired	360	5.0	2,267	7.5
**Level of education**				
No formal education	3,147	73.6	13,241	55.1
Upto primary	1,280	17.9	6,110	23.2
Secondary and above	598	8.5	6,261	21.8
**Social participation**				
Socially active	4,553	90.1	23,665	91.5
Socially inactive	472	9.9	1,947	8.5
**Importance of religion**				
Not important	992	29.7	5,016	20.5
Very important	4,033	70.3	20,596	79.5
**Living arrangement satisfaction**				
Satisfied	4,040	74.6	19,956	74.9
Neutral	817	20.6	4,389	19.4
Not satisfied	168	4.7	1,267	5.7
**Received ill-treatment**				
No	4,894	95.6	24,473	94.8
Yes	131	4.4	1,139	5.2
**Faced discrimination in life**				
No	4,431	83.9	21,535	82.4
Yes	594	16.1	4,077	17.6
**Self-rated health**				
Good	836	15.4	3,488	13.5
Average	3,440	67.0	16,140	63.0
Poor	749	17.6	5,984	23.6
**Chronic morbidity status**				
No condition	3,119	66.6	10,932	45.2
Single condition	1,207	22.7	7,819	29.8
Multiple conditions	699	10.7	6,861	25.0
**Physical activity status**				
Physically inactive	3,403	61.5	19,216	73.3
Physically active	1,622	38.5	6,396	26.7
**Difficulty in ADL**				
No difficulty	4,211	80.5	20,153	76.8
Faces difficulty	814	19.5	5,459	23.2
**Difficulty in IADL**				
No difficulty	3,119	56.4	14,202	52.0
Faces difficulty	1,906	43.6	11,410	48.0
**Covered by health insurance**				
Yes	1,506	29.6	4,964	17.4
No	3,519	70.4	20,648	82.6
**Household MPCE Quintile**				
Poorest	1,452	33.9	4,827	20.7
Poorer	1,081	25.1	5,223	21.4
Middle	948	17.2	5,307	21.0
Richer	844	14.4	5,186	19.8
Richest	700	9.3	5,069	17.1
**Religion of household**				
Hinduism	2,114	79.0	20,345	83.0
Islam	572	2.6	3,050	11.5
Others	2,339	18.3	2,217	5.5
**Place of residence**				
Urban	1,100	10.9	9,331	30.7
Rural	3,925	89.1	16,281	69.3
**Country region**				
Southern	774	8.9	6,592	23.5
Northern	371	13.4	7,417	26.2
Central	395	18.4	1,651	7.7
Western	738	22.0	3,440	16.8
Eastern	562	25.7	5,057	23.6
orth-eastern	2,185	11.6	1,455	2.2
**Overall**	**5,025**	**100**	**25,612**	**100**

(a) *N* Sample size, *WC_%* Weighted column percentage; (b) *ST* Scheduled Tribe, *ADL* Activities of daily living, *IADL* Instrumental activities of daily living, *MPCE* Monthly per capita consumption expenditure

**Fig. 1:**
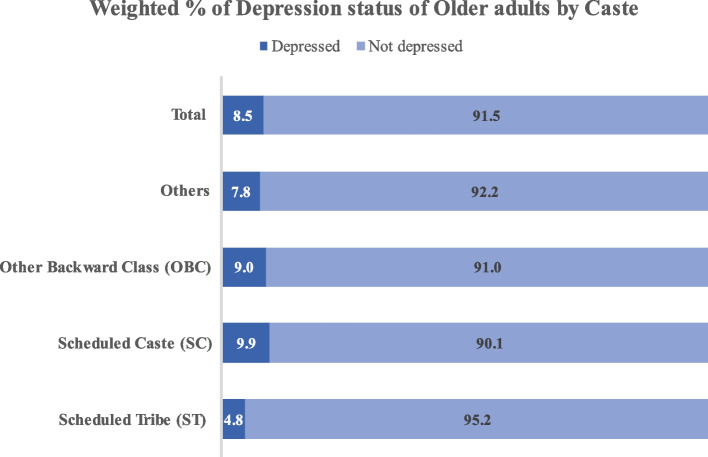
Major depression status of older adults by social groups in India 2017–18

Figure 1 reveals the prevalence of depression among older adults by social groups in India. The highest prevalence of major depression was found among older adults from SC (9.9%) followed by OBC (9.0), Others (7.8%) and ST (4.8%) groups.

### Bivariate analysis

Table 2 depicts the bivariate association between depression and socio-demographic, health-related, and household characteristics of ST and Non-ST older adults in India.

**Table 2:** Bivariate association between depression and socio-demographic, health-related, and household characteristics of ST and Non-ST older adults in India 2017–18

Characteristics	ST older adults (60 + years)				Non-ST older adults (60 + years)			
	**Total**	**Depression**			**Total**	**Depression**		
	**N**	**N**	**WR %**	**Chi2 (** ***p*** **-value)**	**N**	**N**	**WR %**	**Chi2 (** ***p*** **-value)**
***Socio-demographic characteristics***								
**Age group**								
Oldest-old	530	30	8.9	10.82 (0.004)	2,597	214	10.9	1.37 (0.502)
Old-old	1,384	48	6.7		7,459	568	8.3	
Young-old	3,111	90	3.5		15,556	1,180	8.8	
**Gender**								
Male	2,371	74	4.0	0.68 (0.407)	12,311	843	7.7	22.14 (< 0.001)
Female	2,654	94	5.5		13,301	1,119	9.9	
**Current marital status**								
Currently married	3,149	74	3.5	25.75 (< 0.001)	16,367	1,113	8.0	47.43 (< 0.001)
Currently not married	1,876	94	7.0		9,245	849	10.2	
**Status of work**								
Never worked	1,096	32	4.3	18.83 (< 0.001)	7,444	538	7.6	36.98 (< 0.001)
Currently not working	1,605	79	6.1		8,976	801	10.5	
Currently working	1,964	47	4.0		6,925	493	8.3	
Retired	360	10	4.6		2,267	130	7.8	
**Level of education**								
No formal education	3,147	121	5.0	6.55 (0.038)	13,241	1,149	10.0	54.15 (< 0.001)
Upto primary	1,280	32	5.3		6,110	457	8.8	
Secondary and above	598	15	2.4		6,261	356	6.1	
**Social participation**								
Socially active	4,553	147	4.4	1.97 (0.160)	23,665	1,715	8.5	75.24 (< 0.001)
Socially inactive	472	21	8.6		1,947	247	13.0	
**Importance of religion**								
Not important	992	36	4.5	0.31 (0.576)	5,016	346	7.3	5.12 (0.024)
Very important	4,033	132	5.0		20,596	1,616	9.3	
**Living arrangement satisfaction**								
Satisfied	4,040	105	3.9	68.68 (< 0.001)	19,956	1,207	7.0	525.09 (< 0.001)
Neutral	817	40	6.2		4,389	470	11.2	
Not satisfied	168	23	13.5		1,267	285	25.2	
**Received ill-treatment**								
No	4,894	158	4.5	7.66 (0.006)	24,473	1,696	8.1	415.01 (< 0.001)
Yes	131	10	11.0		1,139	266	23.3	
**Faced discrimination in life**								
No	4,431	128	4.4	23.96 (< 0.001)	21,535	1,355	7.4	358.11 (< 0.001)
Yes	594	40	7.1		4,077	607	15.5	
***Health-related characteristics***								
**Self-rated health**								
Good	836	10	1.6	106.25 (< 0.001)	3,488	125	4.2	524.26 (< 0.001)
Average	3,440	87	3.9		16,140	976	6.7	
Poor	749	71	11.0		5,984	861	17.2	
**Chronic morbidity status**								
No condition	3,119	74	3.3	24.14 (< 0.001)	10,932	688	7.5	78.05 (< 0.001)
Single condition	1,207	58	6.6		7,819	594	8.6	
Multiple conditions	699	36	10.5		6,861	680	11.6	
**Physical activity status**								
Physically inactive	3,403	112	4.6	0.08 (0.766)	19,216	1,457	8.9	0.66 (0.414)
Physically active	1,622	56	5.2		6,396	505	8.9	
**Difficulty in ADL**								
No difficulty	4,211	94	3.5	99.29 (< 0.001)	20,153	1,216	6.8	353.67 (< 0.001)
Faces difficulty	814	74	10.3		5,459	746	15.6	
**Difficulty in IADL**								
No difficulty	3,119	59	3.2	53.62 (< 0.001)	14,202	710	5.7	319.16 (< 0.001)
Faces difficulty	1,906	109	6.9		11,410	1,252	12.3	
**Covered by health insurance**								
Yes	1,506	60	5.1	2.73 (0.098)	4,964	290	6.1	28.78 (< 0.001)
No	3,519	108	4.7		20,648	1,672	9.4	
***Household characteristics***								
**Household MPCE Quintile**								
Poorest	1,452	52	3.7	9.86 (0.043)	4,827	404	9.6	17.56 (0.002)
Poorer	1,081	26	4.1		5,223	397	8.1	
Middle	948	27	6.0		5,307	341	8.1	
Richer	844	28	5.4		5,186	401	8.9	
Richest	700	35	7.8		5,069	419	9.8	
**Religion of household**								
Hinduism	2,114	103	5.4	29.91 (< 0.001)	20,345	1,546	8.7	3.38 (0.184)
Islam	572	20	2.9		3,050	257	9.6	
Others	2,339	45	2.7		2,217	159	9.1	
**Place of residence**								
Urban	1,100	31	5.3	1.20 (0.273)	9,331	550	6.3	64.72 (< 0.001)
Rural	3,925	137	4.8		16,281	1,412	10.0	
**Country region**								
Southern	774	20	1.5	41.41 (< 0.001)	6,592	365	5.7	134.20 (< 0.001)
Northern	371	23	8.5		7,417	653	10.5	
Central	395	22	4.0		1,651	208	17.2	
Western	738	35	6.5		3,440	260	7.7	
Eastern	562	28	4.1		5,057	410	8.6	
North-eastern	2,185	40	2.8		1,455	66	6.8	
**Overall**	**5,025**	**168**	**4.8**		**25,612**	**1,962**	**8.9**	

(a) *N* Sample size, *WR_%* Weighted row percentage, *Chi* ^*2*^Chi-square test statistic; (b) *ST* Scheduled Tribe, *ADL* Activities of daily living, *IADL* Instrumental activities of daily living, *MPCE* Monthly per capita consumption expenditure

**Fig. 2:**
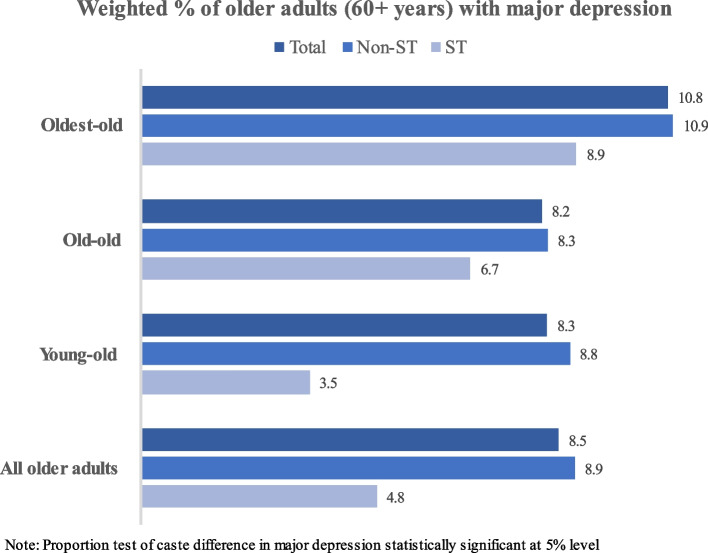
Weighted percentage of the total, Non-ST and ST older adults with major depression in India 2017–18

Figure 2 represents the weighted percentage of the total, Non-ST and ST older adults with major depression in India. It was found that in all the age groups, older adults from non-ST groups had higher prevalence of major depression.

### Multivariate analysis

Table 3 represents the multivariate association between depression and socio-demographic, health-related and household characteristics of ST and Non-ST older adults in India. The odds of depression were higher among young-old in compairison to the odds of depression among oldest old in non-ST population (OR: 1.51; CI: 1.27–1.80). Among the ST (OR: 1.97; CI: 1.36—2.87) and non-ST (OR: 1.26; CI: 11.13—1.41) population, the odds of depression among currently non-married older adults were higher in reference to the odds to depression among currently married older adults. The likelihood of depression was high among currently working older adults in reference to older adults who never worked among non-ST population (OR: 1.29; CI: 1.10—1.52). The likelihood of depression was high among socially inactive older adilts in comparison to older adults who were socially active (OR: 1.45; CI: 1.24—1.70) in non-ST population. In the non-ST population the odds of depression were higher among older adults for whom religion was very important in comparison to the odds of depression among older adults for whom religion was not important (OR: 1.34; CI: 1.17—1.53). The likelihood of depression was high among older adults who were not satisfied with their current living arrangement (OR: 2.55; CI: 2.17—3.01), received ill treatment (OR: 2.02; CI: 1.70—2.40) and faced discrimination in life (OR: 1.68; CI: 1.49—1.90) in reference to their counterparts in both ST and non-ST population. Among the ST and non-ST population the likelihood of depression was high among older adults with poor SRH, multiple morbidity status and difficulty in ADL. The older adults who were physically active had higher odds of depression in reference to the odds of depression among older adults who were physically inactive in ST (OR: 1.86; CI: 1.24—2.79) and non-ST population (OR: 1.20; CI: 1.06—1.35).

**Table 3:** Multivariate association between depression and socio-demographic, health-related and household characteristics of ST and Non-ST older adults in India 2017–18

Characteristics	Depression among older adults (60 + years)			
	**ST**		**Non-ST**	
	**OR**	**95% CI**	**OR**	**95% CI**
***Socio-demographic characteristics***				
**Age group**				
Oldest-old	(Ref)		(Ref)	
Old-old	0.74	(0.44—1.23)	1.16*	(0.98—1.39)
Young-old	0.85	(0.51—1.42)	1.51***	(1.27—1.80)
**Gender**				
Male	(Ref)		(Ref)	
Female	0.76	(0.51—1.13)	1.10	(0.97—1.25)
**Current marital status**				
Currently married	(Ref)		(Ref)	
Currently not married	1.97***	(1.36—2.87)	1.26***	(1.13—1.41)
**Status of work**				
Never worked	(Ref)		(Ref)	
Currently not working	1.25	(0.77—2.03)	1.24***	(1.09—1.42)
Currently working	0.98	(0.55—1.75)	1.29***	(1.10—1.52)
Retired	0.92	(0.39—2.16)	1.24*	(0.99—1.57)
**Level of education**				
No formal education	(Ref)		(Ref)	
Upto primary	0.90	(0.57—1.42)	1.10	(0.97—1.26)
Secondary and above	1.17	(0.59—2.33)	1.06	(0.91—1.24)
**Social participation**				
Socially active	(Ref)		(Ref)	
Socially inactive	1.00	(0.59—1.72)	1.45***	(1.24—1.70)
**Importance of religion**				
Not important	(Ref)		(Ref)	
Very important	1.34	(0.89—2.03)	1.34***	(1.17—1.53)
**Living arrangement satisfaction**				
Satisfied	(Ref)		(Ref)	
Neutral	1.50**	(1.00—2.24)	1.55***	(1.37—1.75)
Not satisfied	3.09***	(1.78—5.35)	2.55***	(2.17—3.01)
**Received ill-treatment**				
No	(Ref)		(Ref)	
Yes	1.28	(0.62—2.66)	2.02***	(1.70—2.40)
**Faced discrimination in life**				
No	(Ref)		(Ref)	
Yes	1.58**	(1.05—2.39)	1.68***	(1.49—1.90)
***Health-related characteristics***				
**Self-rated health**				
Good	(Ref)		(Ref)	
Average	1.64	(0.83—3.23)	1.44***	(1.19—1.76)
Poor	3.82***	(1.85—7.89)	2.83***	(2.29—3.48)
**Chronic morbidity status**				
No condition	(Ref)		(Ref)	
Single condition	1.80***	(1.23—2.64)	1.15**	(1.02—1.30)
Multiple conditions	1.67**	(1.03—2.71)	1.44***	(1.27—1.63)
**Physical activity status**				
Physically inactive	(Ref)		(Ref)	
Physically active	1.86***	(1.24—2.79)	1.20***	(1.06—1.35)
**Difficulty in ADL**				
No difficulty	(Ref)		(Ref)	
Faces difficulty	2.67***	(1.80—3.97)	1.51***	(1.34—1.69)
**Difficulty in IADL**				
No difficulty	(Ref)		(Ref)	
Faces difficulty	1.30	(0.88—1.93)	1.54***	(1.38—1.73)
**Covered by health insurance**				
Yes	(Ref)		(Ref)	
No	0.67**	(0.47—0.95)	1.26***	(1.10—1.44)
***Household characteristics***				
**Household MPCE Quintile**				
Poorest	(Ref)		(Ref)	
Poorer	0.69	(0.41—1.13)	0.99	(0.85—1.16)
Middle	0.94	(0.56—1.56)	0.87*	(0.74—1.01)
Richer	1.09	(0.65—1.83)	1.08	(0.92—1.26)
Richest	2.09***	(1.26—3.47)	1.18**	(1.01—1.38)
**Religion of household**				
Hinduism	(Ref)		(Ref)	
Islam	0.93	(0.46—1.89)	1.02	(0.88—1.19)
Others	0.60*	(0.36—1.02)	1.02	(0.85—1.22)
**Place of residence**				
Urban	(Ref)		(Ref)	
Rural	0.90	(0.56—1.45)	1.31***	(1.16—1.47)
**Country region**				
Southern	(Ref)		(Ref)	
Northern	2.81***	(1.38—5.69)	1.97***	(1.70—2.28)
Central	2.91***	(1.33—6.36)	2.94***	(2.41—3.58)
Western	1.72	(0.84—3.52)	1.81***	(1.52—2.16)
Eastern	2.65**	(1.25—5.61)	1.56***	(1.33—1.83)
North-eastern	1.41	(0.65—3.05)	1.07	(0.81—1.41)
**Analytical sample size**	**5025**		**25,612**	

(a) *OR* Odds Ratio, *CI* Confidence Interval; (b) Statistical significance denoted by asterisks where *p-value < 0.1, **p-value < 0.05 and ***p-value < 0.01; (c) *ADL* Activities of daily living, *IADL* Instrumental activities of daily living, *MPCE* Monthly per capita consumption expenditure

### Decomposing the caste differentials in depression

Table 4 reveals the overall decomposition of differential in depression among older adults from ST and non-ST groups in India. It was found that differences in effects (C) account for 56.2% of the observed social group differences in the prevalence of major depression. However, 43.8% of the differentials in major depression among ST and non-ST groups was explained by differences in compositional characteristics (E).

**Table 4:** Overall decomposition of differential in depression among ST and non-ST older adults in India 2017–18

Component	Differential in depression among older adults		
	**Coefficient**	**95% CI**	**Percent**
**Explained difference (E)**	0.01868***	(0.01388, 0.02348)	43.8
**Unexplained difference (C)**	0.02400***	(0.01682, 0.03118)	56.2
**Raw difference (R)**	0.04268***	(0.03696, 0.04839)	

(a) *CI* Confidence Interval; (b) Statistical significance denoted by asterisks where *p-value < 0.1, **p-value < 0.05 and ***p-value < 0.01

Table 5 represents the detailed decomposition (with normalized coefficients) of ST-non-ST differential in depression among older adults in India. Differences due to characteristics (E) is explained at first. It was revealed that if the regional differences were minimized, it would decrease the gap in major depression among ST and non-ST groups by about 19.6%. Similarly, an equal level of self-rated health and chronic conditions among ST and non-ST would result in reduced gap in major depression by almost 9.6% and 7.9%, respectively. Additionally, equal IADL and ADL difficulty among ST and non-ST older adults would result in reduced gap in major depression by about 3.8% and 3%, respectively. Moreover, if health insurance coverage is equalized among older adults from ST and non-ST populations, it would decrease the gap in major depression by about 3.4%.

**Table 5:** Detailed decomposition (with normalized coefficients) of the differential in depression among older adults in India 2017–18

Characteristics	Differential in depression among older adults					
	**Difference due to Characteristics (E)**			**Difference due to Coefficients (C)**		
	**Coefficient**	**95% CI**	**Percent**	**Coefficient**	**95% CI**	**Percent**
***Socio-demographic characteristics***						
**Age group**						
Oldest-old	0.00008***	(0.00003, 0.00013)	0.2	-0.00103**	(-0.00204, -0.00002)	-2.4
Old-old	-0.00004	(-0.00014, 0.00005)	-0.1	0.00081	(-0.00112, 0.00274)	1.9
Young-old	-0.00018***	(-0.00024, -0.00011)	-0.4	0.00375*	(-0.00062, 0.00813)	8.8
**Gender**						
Male	-0.00003	(-0.00007, 0.00001)	-0.1	-0.00230*	(-0.00491, 0.00030)	-5.4
Female	-0.00003	(-0.00007, 0.00001)	-0.1	0.00259*	(-0.00034, 0.00552)	6.1
**Current marital status**						
Currently married	-0.00012***	(-0.00018, -0.00006)	-0.3	0.00367**	(0.00055, 0.00679)	8.6
Currently not married	-0.00012***	(-0.00018, -0.00006)	-0.3	-0.00226**	(-0.00417, -0.00034)	-5.3
**Status of work**						
Never worked	-0.00076***	(-0.00123, -0.00028)	-1.8	-0.00084	(-0.00318, 0.00151)	-2.0
Currently not working	0.00007	(-0.00007, 0.00022)	0.2	-0.00129	(-0.00399, 0.00142)	-3.0
Currently working	-0.00058	(-0.00133, 0.00016)	-1.4	0.00134	(-0.00250, 0.00519)	3.1
Retired	0.00005	(-0.00012, 0.00021)	0.1	0.00030	(-0.00083, 0.00143)	0.7
**Level of education**						
No formal education	0.00035	(-0.00017, 0.00087)	0.8	-0.00057	(-0.00595, 0.00480)	-1.3
Upto primary	-0.00005	(-0.00014, 0.00004)	-0.1	0.00114	(-0.00106, 0.00335)	2.7
Secondary and above	0.00004	(-0.00069, 0.00078)	0.1	-0.00042	(-0.00181, 0.00097)	-1.0
**Social participation**						
Socially active	-0.00020***	(-0.00029, -0.00011)	-0.5	-0.00439	(-0.01115, 0.00236)	-10.3
Socially inactive	-0.00020***	(-0.00029, -0.00011)	-0.5	0.00049	(-0.00026, 0.00124)	1.1
**Importance of religion**						
Not important	0.00001***	(0.00000, 0.00001)	0.0	0.00001	(-0.00110, 0.00112)	0.0
Very important	0.00001***	(0.00000, 0.00001)	0.0	-0.00003	(-0.00467, 0.00460)	-0.1
**Living arrangement satisfaction**						
Satisfied	0.00069***	(0.00053, 0.00085)	1.6	0.00112	(-0.00451, 0.00675)	2.6
Neutral	-0.00001	(-0.00006, 0.00003)	0.0	0.00036	(-0.00090, 0.00161)	0.8
Not satisfied	0.00046***	(0.00033, 0.00059)	1.1	-0.00012	(-0.00044, 0.00020)	-0.3
**Received ill-treatment**						
No	0.00039***	(0.00027, 0.00050)	0.9	-0.00591	(-0.01576, 0.00394)	-13.8
Yes	0.00039***	(0.00027, 0.00050)	0.9	0.00015	(-0.00010, 0.00041)	0.4
**Faced discrimination in life**						
No	0.00064***	(0.00045, 0.00083)	1.5	-0.00074	(-0.00580, 0.00432)	-1.7
Yes	0.00064***	(0.00045, 0.00083)	1.5	0.00010	(-0.00056, 0.00075)	0.2
***Health-related characteristics***						
**Self-rated health**						
Good	0.00084***	(0.00057, 0.00110)	2.0	0.00062	(-0.00139, 0.00262)	1.4
Average	0.00033**	(0.00006, 0.00061)	0.8	0.00030	(-0.00493, 0.00554)	0.7
Poor	0.00290***	(0.00223, 0.00356)	6.8	-0.00061	(-0.00183, 0.00060)	-1.4
**Chronic morbidity status**						
No condition	0.00196***	(0.00106, 0.00286)	4.6	0.00328	(-0.00087, 0.00742)	7.7
Single condition	-0.00011	(-0.00038, 0.00016)	-0.3	-0.00160**	(-0.00317, -0.00002)	-3.7
Multiple conditions	0.00153***	(0.00090, 0.00217)	3.6	0.00019	(-0.00094, 0.00132)	0.4
**Physical activity status**						
Physically inactive	-0.00039***	(-0.00066, -0.00011)	-0.9	0.00400**	(0.00026, 0.00774)	9.4
Physically active	-0.00039***	(-0.00066, -0.00011)	-0.9	-0.00184**	(-0.00357, -0.00012)	-4.3
**Difficulty in ADL**						
No difficulty	0.00063***	(0.00042, 0.00085)	1.5	0.00630***	(0.00189, 0.01071)	14.8
Faces difficulty	0.00063***	(0.00042, 0.00085)	1.5	-0.00129***	(-0.00220, -0.00039)	-3.0
**Difficulty in IADL**						
No difficulty	0.00083***	(0.00058, 0.00108)	1.9	-0.00137	(-0.00477, 0.00202)	-3.2
Faces difficulty	0.00083***	(0.00058, 0.00108)	1.9	0.00087	(-0.00128, 0.00302)	2.0
**Covered by health insurance**						
Yes	0.00074***	(0.00028, 0.00121)	1.7	-0.00251***	(-0.00401, -0.00101)	-5.9
No	0.00074***	(0.00028, 0.00121)	1.7	0.00589***	(0.00237, 0.00942)	13.8
***Household characteristics***						
**Household MPCE Quintile**						
Poorest	0.00011	(-0.00050, 0.00071)	0.3	0.00045	(-0.00198, 0.00289)	1.1
Poorer	0.00002	(-0.00005, 0.00009)	0.0	0.00246**	(0.00034, 0.00458)	5.8
Middle	-0.00018***	(-0.00030, -0.00007)	-0.4	-0.00011	(-0.00195, 0.00173)	-0.3
Richer	0.00012	(-0.00008, 0.00033)	0.3	0.00021	(-0.00142, 0.00183)	0.5
Richest	0.00052***	(0.00017, 0.00088)	1.2	-0.00190***	(-0.00319, -0.00061)	-4.4
**Religion of household**						
Hinduism	-0.00035	(-0.00225, 0.00156)	-0.8	-0.00231	(-0.00594, 0.00132)	-5.4
Islam	0.00000	(-0.00005, 0.00006)	0.0	-0.00033	(-0.00180, 0.00113)	-0.8
Others	-0.00015	(-0.00305, 0.00276)	-0.3	0.00397	(-0.00121, 0.00914)	9.3
**Place of residence**						
Urban	-0.00118***	(-0.00173, -0.00062)	-2.8	-0.00109	(-0.00255, 0.00036)	-2.6
Rural	-0.00118***	(-0.00173, -0.00062)	-2.8	0.00387	(-0.00129, 0.00904)	9.1
**Country region**						
Southern	-0.00304***	(-0.00406, -0.00202)	-7.1	0.00075	(-0.00137, 0.00288)	1.8
Northern	0.00264***	(0.00146, 0.00382)	6.2	-0.00033	(-0.00121, 0.00055)	-0.8
Central	-0.00052***	(-0.00066, -0.00039)	-1.2	0.00041	(-0.00059, 0.00140)	1.0
Western	-0.00010*	(-0.00020, 0.00000)	-0.2	0.00092	(-0.00066, 0.00250)	2.2
Eastern	-0.00016	(-0.00074, 0.00041)	-0.4	-0.00102	(-0.00224, 0.00019)	-2.4
North-eastern	0.00953***	(0.00570, 0.01336)	22.3	-0.00112	(-0.00712, 0.00489)	-2.6
**Constant**				0.01102	(-0.00570, 0.02773)	25.8

(a) *CI* Confidence interval; (b) Statistical significance denoted by asterisks where *p-value < 0.1, **p-value < 0.05 and ***p-value < 0.01; (c) *ADL* Activities of daily living, *IADL* Instrumental activities of daily living, *MPCE* Monthly per capita consumption expenditure

Also, findings from differences due to coefficients (C) revealed that currently married status (8.6%) and being socially active (10.3%) significantly contributed to the ST-non-ST inequality in major depression in this study. Similarly, it was found that if older adults from non-ST population had the same status of ADL as of older adults from ST population, it would decrease the gap in major depression by about 11.8%. Similarly, if older adults from ST group had the same coverage of health insurance as of non-ST group, this would decrease the gap in major depression by almost 7.9%. Additionally, if older adults from non-ST population had same level of physical activity as of older adults from ST population, it would decrease the gap in major depression by about 5.7%.

## Discussion

The present study investigated the differentials in the prevalence and determinants of major depression among ST and non-ST populations and decomposed the key socio-demographic, health, and household factors that accounted for the ST/non-ST-based inequalities in major depression among older adults in India by utilizing large-scale nationally representative survey data. The prevalence of major depression in the ST population was lower than the non-ST population (4.8% vs. 8.9%). Multiple studies based on indigenous tribal populations in India reported a lower prevalence of severe depression than in our study [30, 31]. For instance, a study conducted in the tribal community living in the Phek district of Nagaland, India, found that nearly 1.2% of older adults were experiencing depression [30]. Another recent study based on the tribal population of the Mysuru district in Karnataka, India, found a prevalence of severe depression of 3.5% among older adults [31]. In contrast, several other small-scale studies have reported a greater prevalence of depression in tribal older adults in India [32, 33]. The inconsistencies in the prevalence of depression may be partially due to variations in geographical areas, sample measures, and measurement tools.

Results from multivariable analysis demonstrated that physically active older adults had significantly higher odds of major depression than their physically inactive counterparts, which is inconsistent with most of the previous studies [34, 35]. This inconsistent relationship may be due to different definitions of physical activeness in other studies. This study did not find any association between various socio-demographic factors such as age and working status and major depression in the tribal population. On the other hand, in the non-tribal group, increasing age and current working status were significantly positively related to major depression. Moreover, other socio-demographic factors such as gender and educational attainments were unrelated to major depressive disorders in tribal and non-tribal populations.

There are possible explanations for the lower depressive symptoms in the ST population than in the non-ST population. Many studies have reported that tribal populations have a lower level of non-communicable chronic conditions [36], more physically active lifestyle [37, 38], healthy diet and lifestyles [39, 40], and belief in spiritual rituals that can play a significant role in healing and wellness [41]. On the other hand, these behaviors like physical activeness, lesser chronic conditions, healthy diet patterns, active lifestyles, and spiritual orientations have been associated with reduced depressive symptoms [42–45]. Therefore, it could be possible that such behaviors in tribal populations could effectively enhance mental health.

The decomposition analysis showed that the selected explanatory variables explained nearly 43.8% of the ST/non-ST inequality in major depression in the present study. The major contributors to the explained component were regional differences, self-rated health status, and chronic conditions. Around 19.6% of inequalities in major depression were attributed to regional differences; this is due to the dissimilarities in the distribution of ST and non-ST populations across different regions. For instance, as our data showed, the ST population is majorly concentrated in the north-eastern region, while a greater proportion of the non-ST population is concentrated in the northern and western regions of India. Differentials in self-rated health contributed to nearly 10% of the caste inequalities in the prevalence of major depression, resulting from the fact that a greater proportion of older adults in the non-ST group reported poor self-rated health than older adults in the ST group (23.4% vs. 14.9%). These findings are consistent with the other studies suggesting that poor self-rated health [46–48] is linked to having major depressive disorders in older persons.

Importantly, age, marital status and social participation contributed significantly to the ST-Non-ST differentials in major depression which may imply the greater buffering effects of increasing age, presence of structural support and participation in social activities on reducing major depression among older adults from ST category than those from non-ST group. Consistent with our findings, numerous studies have reported that widowhood and lack of social support can elevate stress, therefore, leading to higher levels of depression [49–52].

Nearly 8% of the inequalities in major depression were attributed to the differentials in the prevalence of chronic conditions among ST and non-ST populations; this is because a greater proportion of older adults in the non-ST group reported having multiple chronic conditions than older adults in the ST group (26.8% vs. 13.9%). Although studies related to chronic conditions in the tribal population in India are limited, the available studies reported a severe lack of awareness of chronic diseases among the tribal population in India [53]. However, in comparison to the general population, tribal population are expected to have lesser chances of cardiovascular diseases as they are physically active and are more prone to have plant-based foods. Another important contributor to the ST-non-ST differential in major depression was ADL and IADL functional difficulty. The findings are also in concordance with previous studies on the linkage between multiple chronic conditions [54, 55], functional limitations [56] and major depression.

There are some strengths and limitations of this study worth mentioning. The utilization of a large sample from a national representative survey in deriving the estimates is the potential strength of the study. An additional strength of the study is using the CIDI-SF scale for deriving reliable and valid estimates for major depression. There are potential limitations to the study. Firstly, our explanatory variables, such as self-reporting of the diagnosed chronic conditions, can be under-reported either due to unawareness about the chronic condition among the older adults or by recall bias. Moreover, the responses to the variables like self-rated health may be biased because individuals use response scales in systematically different ways. Finally, our results are based on the cross-sectional data; therefore, establishing a causal direction of the association of explanatory variables with major depression is not possible.

## Conclusions

The present study found a greater prevalence of major depression in older adults in the ST group than in the non-ST group. A greater percentage of the ST/non-ST inequality in major depression, advantageous to tribal population in the present study was explained by age, marital status, and social participation, suggesting further research on the coping mechanisms of mental illnesses among the indigenous population in India. The ST-non-ST differentials in the major depression were also attributed to regional differences, self-rated health status, and chronic conditions, which need to be explored in future research.

## Data Availability

The study uses secondary data which is available on reasonable request through https://www.iipsindia.ac.in/content/lasi-wave-i
